# Antioxidant Activity of Hydrogen Water Mask Pack Composed of Gel-Type Emulsion and Hydrogen Generation Powder

**DOI:** 10.3390/ijms21249731

**Published:** 2020-12-20

**Authors:** Hye-Jin Kwon, Sang-Beom Han, Kyung-Won Park

**Affiliations:** 1 Department of Chemical Engineering, Soongsil University, Seoul 06978, Korea; kwonhj0070@ssu.ac.kr (H.-J.K.); sciresb@hanmail.net (S.-B.H.); 2 BoyazEnergy, 165 Gasandigital 2-ro, Geumcheon-gu, Seoul 08504, Korea

**Keywords:** hydrogen generation powder, hydrogen water face mask, antioxidant activity

## Abstract

In this study, hydrogen generation powder samples were prepared using zinc carbonate as a precursor, at a temperature varying from 400 to 700 °C in H_2_ atmosphere, and were characterized in terms of antioxidant activity. The concentration of dissolved hydrogen obtained by the powder samples was measured using a dissolved hydrogen meter as a function of time. In addition, the antioxidant activity of the samples was evaluated based on the Oyaizu’s method, removal rate of ·OH radicals, and ferric reducing antioxidant power. Finally, the hydrogen mask pack was fabricated using the hydrogen generation powder sample and gel-type emulsion. In the clinical test on the mask pack, the effect of the mask on skin aging was characterized and compared to that of a commercial sample. The skin densities of the participants in the experimental group and the control group increased by 18.41% and 9.93% after 4 weeks, respectively. The improved skin density of the participants who used the hydrogen mask pack in the experimental group, might be attributed to the recovery effect of the hydrogen molecule in the mask pack on the denatured thick skin layer.

## 1. Introduction

In the 21th century, the era of health care has made life prolongation feasible, beyond the treatment of the diseases. Aging and diseases are universal and essential phenomena, which have specific causes and treatments depending on the symptoms [1]. However, Gianluca et al. showed that the main causes of death and diseases might be significantly associated with the free oxygen radicals [2]. Naturally, the human organism after the age of 40 cannot generate superoxide dismutase (SOD) as an antioxidant enzyme, which can remove free oxygen radicals [3]. In general, vitamins A, C, and E can help remove free oxygen radicals in the human body [4]. Furthermore, hydrogen can be an antioxidant candidate because of its excellent antioxidant activity to selectively remove free oxygen radicals, and its accessibility to human organisms and brain cells [5]. In particular, recently, hydrogen water, i.e., water that contains extra hydrogen gas via several means, has been considered a promising tool because of its various applications in medical treatment, health, and skin care [6].

Cosmetic products in the era of health care have been used not only to protect the skin but also to treat skin problems [7]. Thus, functionally effective materials in cosmetics need to be able to improve percutaneous absorption and stabilization [8]. Among these functional cosmetics, mask packs, as instant beauty products, that can improve human skin density through an instantaneous change, have emerged as popular items [9]. Recently, sheet-type mask packs containing hydrogen water have been on the market. However, hydrogen can be scarce in conventional sheet mask packs or can instantaneously disappear when opening the packaging of mask packs [10]. In this study, we proposed a novel hydrogen water mask pack consisting of hydrogen generation powder and gel-type emulsion. When mixing the powder with the emulsion, high-concentrated hydrogen gas is continuously generated for several minutes to effectively remove oxygen radicals on the skin.

## 2. Results

### 2.1. Characterization of Hydrogen Generation Powder Samples

**Figure 1:**
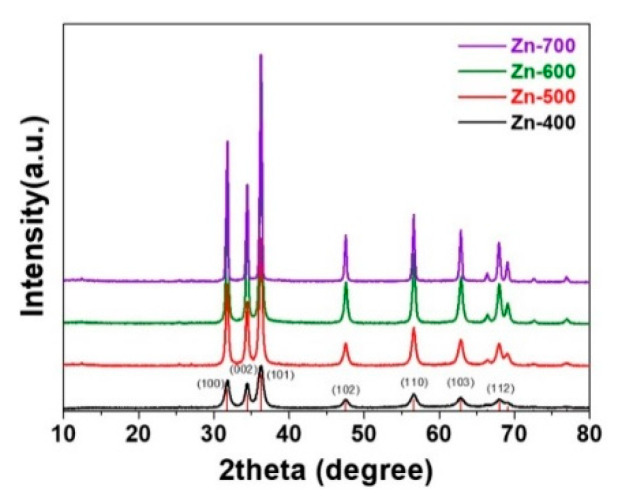
XRD patterns of the as-prepared hydrogen generation powder samples.

Figure 1 shows the XRD patterns of the hydrogen generation powder samples heated in an H_2_ atmosphere at various temperatures. The samples were prepared by heating in an H_2_ atmosphere at 400, 500, 600, and 700 °C, respectively (Zn-400, Zn-500, Zn-600, and Zn-700). All the samples exhibited a zincite (PDF 70-2551) crystalline structure without other phases, with the XRD characteristic peaks at 31.84°, 34.52°, 36.66°, 47.63°, 56.71°, and 62.96°, corresponding to the (100), (002), (101), (102), (110), and (103) planes, planes, respectively. Despite the hydrogen atmosphere heating, no metallic Zn phase was formed. The average crystallite sizes of the Zn-400, Zn-500, Zn-600, and Zn-700 samples, calculated by the Scherrer equation, were 12.5, 20.2, 27.2, and 30.9 nm, respectively. Although, with increasing heating temperature, the crystallite size of the heated samples increased, all the samples were nano-sized powder.

**Figure 2:**
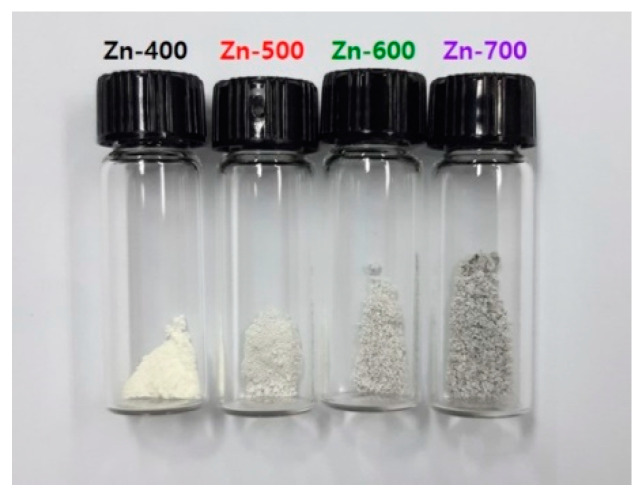
Photo images of the hydrogen generation powder samples prepared with zinc. Carbonate as a precursor at 400, 500, 600, and 700 °C in H_2_ atmosphere.

Figure 2 shows the photo images of the hydrogen generation powder samples heated at various temperatures. The Zn-400, Zn-500, and Zn-600 samples were light yellow whereas the Zn-700 sample was light gray. In general, pure ZnO with a band gap of 3.37 eV is white, whereas all the prepared samples were yellow or gray. Thus, the change in color in the samples heated in H_2_ atmosphere indicates the generation of defect or doping levels in the band gap, forming ZnO_1−x_. The narrow band gap might allow electrons from the valence band to be elevated into the conduction band. The absorbance of blue light in the hydrogen generation powder samples might result in the reflected color yellow or gray. In general, partially reduced ZnO_1−x_ has been prepared by heating Zn metal at 950 °C in the presence of carbon or carbon monoxide [11]. However, in this study, the nano-sized ZnO_1−x_ samples for the hydrogen generation were prepared at relatively low temperatures.

**Figure 3:**
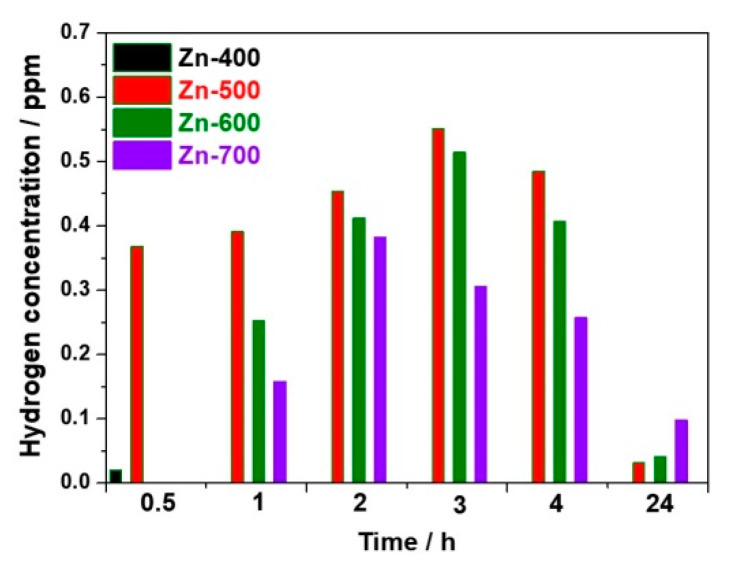
The concentration of hydrogen generated from the powder samples as a. function of time.

Figure 3 compares the hydrogen concentration generated from the hydrogen generation powder samples as a function of time. For the reduced ZnO_1−x_, the hydrogen evolution might occur in the aqueous solution, during complete oxidation of ZnO_1−x_ to ZnO, as follows:

ZnO_1−x_ + xH_2_O → ZnO + xH_2_↑ (1)

Thus, a higher portion of the reduced ZnO_1−x_ might lead to a higher hydrogen evolution, oxidizing ZnO_1−x_ to ZnO. For the Zn-400 sample, the hydrogen concentration was 0.02 ppm after 30 min and, afterward, approached almost zero because of a low reduction portion in ZnO. For the Zn-500 sample, the hydrogen concentration increased to 0.02 and 0.551 ppm after 30 min and 3 h, respectively, and subsequently decreased to 0.031 ppm after 24 h. For the Zn-600 sample, the hydrogen concentration increased to 0.252 and 0.514 ppm after 1 and 3 h, respectively. For the Zn-700 sample, the hydrogen concentration increased to 0.158 and 0.382 ppm after 1 and 2 h, respectively, and, subsequently, was maintained at 0.098 ppm after 24 h. As the heating temperature increased from 500 to 700 °C, the hydrogen generation rate might be slow because of the increased crystallite size of the samples and the initial concentration of hydrogen in the solution might be low. However, the higher heating temperature could lead to the increased portion of ZnO_1−x_, enhancing the hydrogen evolution. Thus, in terms of the crystallite size and degree of reduction along with heating temperature, the Zn-500 sample prepared at a proper heating condition exhibited the best activity for hydrogen generation.

### 2.2. Antioxidant and Antiradiacal Activities of the Hydrogen Generation Powder Samples

**Figure 4:**
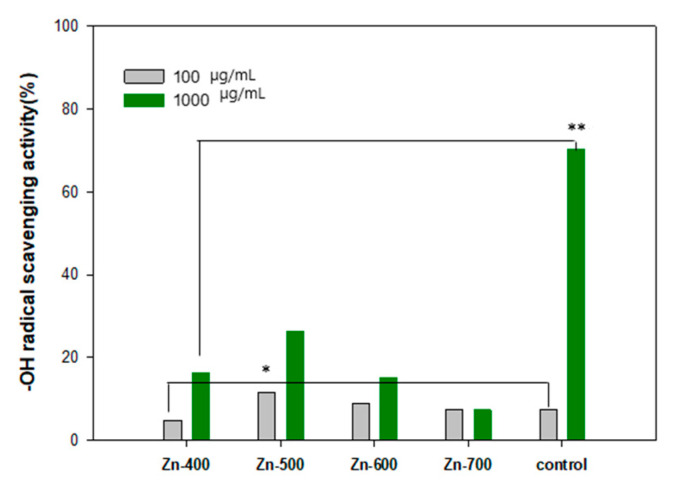
⋅OH radical scavenging activity of each samples. The results are expressed as mean ± S.D from four independent experiments. The level of significance (*). between before and after the utilization is *p* < 0.05. The level of significance (**) between the groups is *p* < 0.01.

The reducing power of the hydrogen generation powder samples was measured using the Oyaizu’s method. The reductants of an antioxidant reduce the complex agent of Fe^3+^/ferricyanide to ferrous compound, resulting in the Perl’s Prussian blue. The degree of the transition of the complex agent of Fe^3+^/ferricyanide to ferrous compound was measured using an absorbance at a wavelength of 700 nm [12]. The high intensity of the absorbance demonstrates the improved reducing power of the hydrogen generation powder. The reducing power of the hydrogen generation powder samples was compared, as shown in Table 1. Compared to a commercial powder sample (Wakans Hydrogen Pack, Japan), the prepared samples showed a comparable reducing power. Among reactive oxygen species (ROS), ⋅OH is known to be the most active hydroxyl radical and can seriously damage biometric molecules. Specifically, the concentration of H_2_O_2_ and ⋅OH on human skin under UV irradiation may increase. The formed ROS is the major reason that can lead to oxidative damages in deoxyribonucleic acid (DNA), protein, cell membrane, and lipoid. Furthermore, skin aging can result from the decomposition of main components (collagen, elastin, and hyaluronic acid) of the skin extracellular matrix by ROS [13,14,15,16]. Hydrogen gas could be generated during the oxidation process of water molecules with the hydrogen generation powder sample. The elimination effect of ⋅OH radical by the hydrogen generation powder samples was compared with a commercial powder sample (Wakans Hydrogen Pack, Japan) (Figure 4). The Zn-500 sample showed the best removal rates of 11.71 ± 0.89% and 26.23 ± 0.45% at the concentration of 100 μg·mL^−1^ and 1000 μg·mL^−1^, respectively.

**Figure 5:**
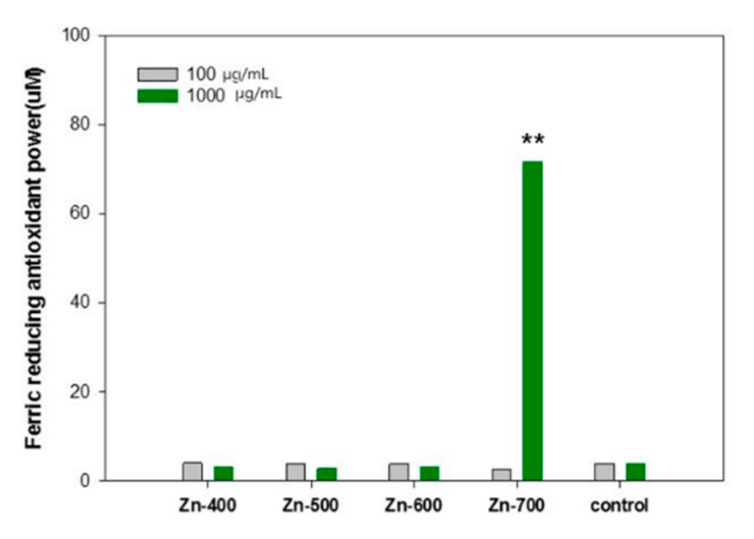
Ferric-reducing antioxidant power of each samples. The results are expressed as mean ± S.D from four independent experiments. The level of significance (**) between the groups is *p* < 0.01.

The reducing power of antioxidants can be evaluated by measuring the reduction in ferric tripyridyltriazine (Fe^3+^-TPTZ) to ferrous tripyridyltriazine (Fe^2+^-TPTZ) in a low pH [17]. The values of the Ferric Reducing Ability of Plasma (FRAP) of the samples were compared (Figure 5). At the concentration of 100 μg·mL^−1^, the FRAP of all the samples was similar. However, at the concentration of 1000 μg·mL^−1^, the Zn-700 sample exhibited significantly high FRAP values of 71.83 ± 0.16 and 90.59 ± 0.29, respectively. Overall, based on Oyaizu’s and FRAP analysis, the hydrogen generation powder samples synthesized using the present preparation method are considered to have an antioxidant effect. It was reported that the hydrogen molecule can reduce hydroxyl radicals (⋅OH) and peroxynitrite (ONOO) by the following mechanism [18,19]:

H_2_ + ⋅OH → H_2_O + H⋅ (2)

H⋅ + O_2_^−^ → HO_2_^−^ (3)

OH has been a well-known trigger of the chain reaction of free radicals and it can cause oxidative damage to biomolecules in cells. Because just a few effective enzymes are able to treat ⋅OH acting as the most active ROS, the hydrogen molecule has been considered as a promising antioxidant [20,21]. In this study, to elaborately evaluate antioxidant and antiradical activities of each samples, the reducing power (Table 1), ⋅OH radical scavenging activity (Figure 4), and ferric-reducing antioxidant power (Figure 5) of each of the samples were measured. In the Table 1, the reducing power is related to electron donation to active oxygen species and free radicals. The Zn-600 and Zn-700 samples showed an improved reducing power in comparison with the control group. In addition, the Zn-400 and Zn-500 samples have a comparable activity to a commercial powder sample. In the comparison of ⋅OH radical scavenging activity (Figure 4), among these hydrogen generation powder samples, the Zn-500 sample exhibited the best activity. The Zn-700 sample showed superior performance in the ferric-reducing antioxidant power test (Figure 5). Based on these essential evaluations for antioxidant activity, the Zn-500 or Zn-700 as a cosmetic powder source could be promising for clinical test. However, eventually, compared to the Zn-700, the Zn-500 sample with a relatively finer powder state owing to its smaller particle size was selected as a cosmetic powder source for a gel-type mask pack prepared with chemical gel and additives.

### 2.3. Clinical Test Hydrogen Mask Packs

**Figure 6:**
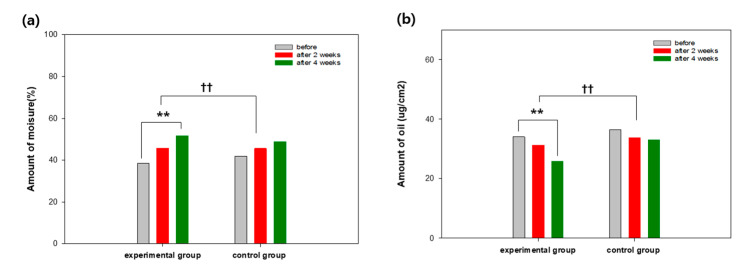
Skin condition changes in (**a**) moisture and (**b**) sebum. The level of significance (**). between before and after the utilization is *p* < 0.01. The level of significance (††) between the groups is *p* < 0.01.

Before the clinical test, the amount of moisture and oil, and the density of the thick skin of the participants were considered to be identical (*p* > 0.1). In the experimental group, the moisture content increased by 19.21% and 34.63% after 2 and 4 weeks, respectively. In the control group, the moisture content increased by 8.94% and 16.51% after 2 and 4 weeks, respectively (Figure 6). For comparison, a commercial product for the control group was utilized. The amount of oil in the experimental group decreased by 8.41% and 24.06% after 2 and 4 weeks, respectively, whereas the amount of oil in the control group decreased by 9.55% after 4 weeks (Figure 6). The dead skin cell layer as a skin outermost layer forms a cornified envelope consisting of multilayered proteins instead of a protoplasm cell composed of polarity lipoid. The cornified envelope is covalently bonded to transglutaminase and contains proteins, where proline is abundant. The outer surface of the cornified envelope is covered with lipoid for its hydrophobicity. The skin of lipoid is formed by a covalent bond between the end of involucrin and ω-hydroxyl ceramide [22]. It was reported that carbonylation protein was found in the dried skin exposed to UV. The portion of the carbonylation protein in the dead skin cell is inversely proportional to the amount of moisture in the dead skin cell, resulting in more dried skin. Thus, suppression of the formation of the carbonylation protein may be effective to maintain moisturized skin. Furthermore, antioxidants can assist in the inhibition of formation of aldehyde compounds generated during peroxidative reaction of lipoid, improving the moisture retention in skin [23,24,25]. In particular, sebum can cause bacterial growth such as P. acne, affecting the condition of skin [26]. Since the amount of sebum in moisture-deficient skin relatively is increased, the decreased amount of sebum might be attributed to the increment of moisture in skin [27].

**Figure 7:**
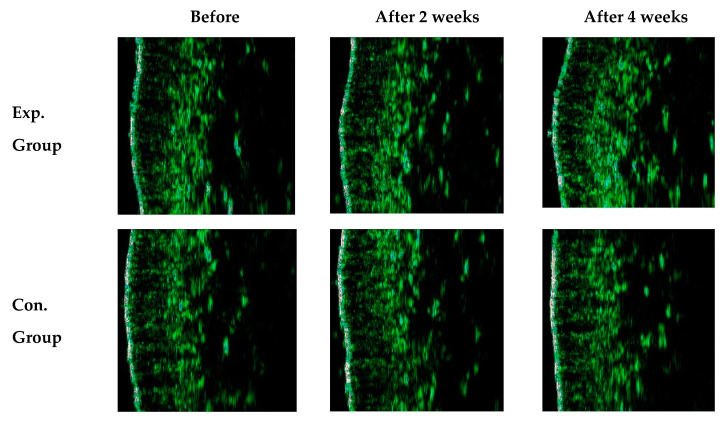
B-scan mode image of skin density before the mask pack utilization and after 2 and 4 weeks.

The density of the thick skin in the experimental group varied by 12.53% and 18.41% after 2 and 4 weeks (*p* < 0.01). Figure 7 shows transition images of skin density of a participant in the experimental group observed by B-scan mode measurement. During the measurement of the skin density, many echoes can occur passing through the inner skin composed of collagen and moisture-rich cellular matrix. The higher echo displays in color order of white-yellow-red-green-blue-black. The skin density of the participant in the experimental group varied by 18.41% after 4 weeks, whereas the skin density of the participant in the control group varied by 9.93% after 4 weeks (*p* < 0.01).

## 3. Discussion

**Figure 8:**
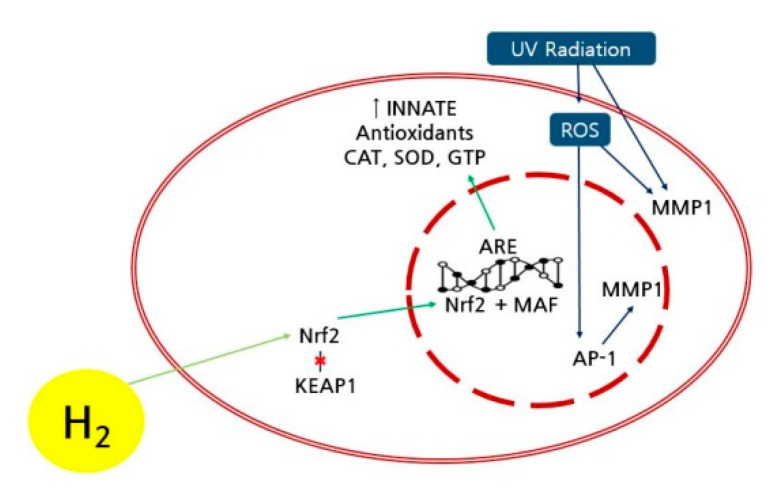
Mechanism of the improved skin density using the hydrogen mask pack. Antioxidant enzymes: catalase (CAT), superoxide dismutase (SOD), and glutathione (GTP)). KEAP 1: Kelch-like Epichlorohydrin-associated protein 1. AP 1: Activator protein 1. ARE: antioxidant response element, MAF: musculo aponeurotic fibrosarcoma.

The extrinsic skin aging can be attributed to ROS, which can oxidize and destroy collagen in the thick skin, and UV irradiation, which can destroy the elastic fiber in the thick skin (Figure 8) [28]. The UV-exposed skin can induce matrix metalloproteinase-1 (MMP-1) or collagenase 1 combined with ROS, resulting in the destruction of the extracellular matrix protein and structural damage of the thick skin [29]. The accumulation of ROS and MMPs can reduce the synthesis of collagen in the skin [30]. Recently, it was reported that hydrogen may suppress the formation of ROS caused by UV and the expression of MMP-1, thus preventing the UV-exposed skin aging [31]. Furthermore, the bridge-building of collagen and elastin during glycation process might accelerate the skin aging with active oxygen species [32]. Since the hydrogen as the smallest molecule can easily permeate the cell membrane and rapidly diffuse into minute organs in the cells, the antioxidant activity of hydrogen can significantly delay the skin aging. Specifically, the nuclear factor erythroid 2-related factor 2 (Nrf2) is a key factor affecting the control of antioxidant activity and constancy, activating the transport path of the Nrf2 by hydrogen molecule [33,34,35]. The activation of Nrf2 can increase the gene expression of antioxidative enzymes such as superoxide dismutase and catalase and protect skin cells including dead skin, fibroblast, and melanocyte from UV-derived oxidative damage and cellular malfunction [36]. It was reported that, specifically, the hydrogen molecule could activate the path of Nrf2 because of the increase in antioxidative enzymes [37]. The dissociation of collagen as a result of skin oxidative damage and aging process can be blocked by the activation of Nrf2 caused by the hydrogen molecule [38]. Thus, the increased skin density of the participant using the hydrogen mask pack in the experimental group can be attributed to the improvement of skin aging in the denatured thick skin layer. Furthermore, hydrogen molecules in disease treatments have been extensively utilized because of genetic modification, antioxidant activity, and anti-inflammatory function [39,40,41]. However, since accurate path and mechanism of skin improvement caused by hydrogen molecule need to be intensively studied, with further research on an optimized concentration of hydrogen molecule and an established method of use in the hydrogen mask pack.

## 4. Materials and Methods

### 4.1. Preparation and Measurement of Hydrogen Generation of Powder Samples

The hydrogen generation powder samples were heated at different temperatures (400 to 700 °C) in an H_2_ gas atmosphere. Zinc carbonate (3 g, JUNSEI Co., Tokyo, Japan) was loaded on a quartz boat and transferred to a tube furnace. Subsequently, the precursor was purged in H_2_ gas for 1 h, heated up to 400, 500, 600, and 700 °C, and maintained for 2 h. The structure of the prepared samples was analyzed using an X-ray diffractometer (XRD, Bruker D2 Phase system) equipped with a Cu K_α_ radiation source of λ = 0.15406 nm and a Ni filter. To measure the concentration of H_2_ in a solution, 0.1 g of the hydrogen generation powder sample was added to 100 mL of de-ionized (DI) water and continuously stirred using a magnetic bar at a rotating speed of 500 rpm. The concentration of dissolved hydrogen generated by the powder sample was measured using a dissolved hydrogen meter (DH30, Clean Instruments Co., Ltd., Shanghai, China). The hydrogen concentration was measured as a function of time after 2 min to stabilize the hydrogen generation.

### 4.2. Evaluation of Antioxidant and Antiradiacal Activities

#### 4.2.1. Measurement of Reducing Properties

The reducing properties of the powder samples were measured using the Oyaizu’s method [42]. The powder samples (0.1 mg) were mixed in 10 mL of distilled water. The powder solution (100 μL) was mixed with 0.2 M sodium phosphate buffer (500 μL, pH 6.6) and 1% potassium ferricyanide (500 μL) at 50 °C for 2 min. Subsequently, 2.5 mL of 10% trichloroacetic acid was added to the solution. The mixed solution was centrifuged at 650 rpm for 10 min. The upper liquid (500 μL) in the centrifuged sample was mixed with DI water (500 μL) and 1% ferric chloride (100 μL). The optical density (OD) of the resulting solution was measured at λ = 700 nm.

#### 4.2.2. Measurement of Removal of ⋅OH Radical

The ⋅OH radical elimination was measured using the 2-deoxyribose oxidation method [43]. The samples (1400 μL) were mixed with 200 μL of 10 mM FeSO_4_⋅7H_2_O-ethylene diamine tetraacetic acid (EDTA), 200 μL of 10 mM 2-deoxyribose, and 200 μL of 10 mM hydrogen peroxide. The mixed solutions were incubated at 37 °C for 4 h. Thiobarbituric acid (1.05, 1 mL) and trichloroacetic acid (2.8%, 1 mL) were added to the incubated solutions and were boiled for 20 min. The OD values of the resulting solutions were measured at λ = 490 nm.

#### 4.2.3. Measurement of Ferric Reducing Antioxidant Power (FRAP)

The powder samples were diluted to 100 and 1000 μg mL^−1^. The FRAP agent was prepared by mixing 300 mM sodium acetate buffer (pH 3.6), 10 mM 2,4,6-tris(2-pyridyl)-s-triazine (TPTZ) and 20 mM FeCl_3_ in 40 mM HCl at the ratio of 10:1:1. The FRAP reagent (2 mL) and DI water (900 μL) were added to the prepared powder sample (100 μL) and stored in the dark for 30 min. The OD values of the resulting solutions were measured at λ = 593 nm. The FRAP of the samples was calculated using FeSO_4_ as a standard [44].

### 4.3. Clinical Test Using Hydrogen Mask Pack

#### 4.3.1. Subjects and Study Design

In this study, the clinical test was performed after the approval (CDIRB-QR-20-057) of the ethics commission of the Korea clinical test institution (COREDER). All the protocols and procedures were carried out according to the declaration of Helsinki. The candidates for participating the clinical test put a hydrogen mask pack patch (size: 3 cm × 3 cm) inside an arm for 24 h. The skin reactivity of the candidates was deciphered using a Draize method (Frosch and Kligman, CTFA guideline) [45]. Then, the skin stimulate index was calculated to evaluate the safety of skin. The final twenty participants for clinical tests were selected on the basis of the skin stimulate index. All the participants in the clinical trial submitted the agreement before the clinical test. Individuals with particular skin symptoms, a similar experience within 3 months, and who were taking drugs, were excluded. The stability test of the prepared hydrogen mask pack for heavy metals, escherichia coli, pseudomonas aeruginosa, and staphylococcus aureus was performed on the basis of the standard in the Korean Food and Drug Administration. The gel-type mask pack was fabricated using hydrogen generation powder (20%) and base gel (glycerin, hydroxyethylcellulose, and cellulose gum), with additives such as Zea mays (corn) starch, Helianthus annuus seed oil, magnesium sulfate, and silica. Before the clinical test, the participants mixed the hydrogen generation powder with the base gel and applied the pack to the face. For comparison, a commercial product (Radian, nh_rinasce_pack01, Japan) was utilized. The main ingredients of the hydrogen powder were sodium hydrogen carbonate, magnesium carbonate, magnesium sulfate, and potassium carbonate. The base gel mainly composed of Zea mays starch, sunflower seed oil, 2, 3-butylene glycol, glycerin, and sodium hyaluronate.

#### 4.3.2. Measurement of Skin Biophysical Parameters

The participants washed their face every morning and applied the prepared and commercial packs to the left and right sides of the faces for 4 weeks. The transition states of the skins were observed and measured before the test, and after 2 and 4 weeks. After washing and 20 min relaxation at a constant temperature (22 ± 2 °C) and relative humidity (50 ± 5%), the moisture, sebum, and thick skin density on the face were measured using a corneometer (CM825, C+K, Köln, Germany), sebumeter (SM810, C+K, Köln, Germany), and skin scanner (tpmGmbH, DUB, Köln, Germany) [46,47]. All the measurements were performed three times and verified using the statistical package for the social science (SPSS) package program (ver. 26, IBM, New York, NY, USA). The comparison of the evaluation items for the masks was carried out using the Wilcoxon signed-rank test. The group homogeneity was confirmed using the Mann–Whitney test (level of significance, *p* > 0.1).

## 5. Conclusions

In summary, the hydrogen generation powder samples were prepared with zinc carbonate as a precursor at temperature varying from 400 to 700 °C in H_2_ atmosphere and were characterized in terms of antioxidant activity. Overall, the antioxidant activity of the hydrogen generation powder samples was superior to that of the commercial sample. In the clinical test using the hydrogen mask pack fabricated using the hydrogen generation powder sample and gel-type emulsion, the skin density of the participant in the experimental group increased by 18.41% after 4 weeks whereas the skin density of the participant in the control group increased by 9.93% after 4 weeks. Consequently, the improved skin density, after the utilization of the hydrogen mask pack in the experimental group, might result from the recovery effect of the hydrogen molecule in the mask pack on the denatured thick skin layer.

## Figures and Tables

**Table 1:** Reducing power of each samples. The results are expressed as mean ± S.D from four independent experiments.

Sample	Optical Density Value@700 nm
Zn-400	0.297 ± 0.001
Zn-500	0.294 ± 0.002
Zn-600	0.308 ± 0.001
Zn-700	0.307 ± 0.001
A commercial sample	0.292 ± 0.001
